# Peripheral endothelial dysfunction is associated with gas exchange inefficiency in smokers

**DOI:** 10.1186/1465-9921-12-53

**Published:** 2011-04-25

**Authors:** Sven Gläser, Anne Obst, Christian F Opitz, Marcus Dörr, Stephan B Felix, Klaus Empen, Henry Völzke, Ralf Ewert, Christoph Schäper, Beate Koch

**Affiliations:** 1 Medical Faculty of the Ernst-Moritz-Arndt University, Department of Internal Medicine B - Cardiology, Intensive Care, Pulmonary Medicine and Infectious Diseases, Friedrich-Loeffler-Str. 23, D-17475 Greifswald, Germany; 2 Institute for Community Medicine, SHIP/Clinical-Epidemiological Research, Walther-Rathenau-Str. 48, 17487 Greifswald, Germany; 3 Department of Cardiology, DRK Kliniken Köpenick, Salvador-Allende-Straße 2-8, D-12559 Berlin, Germany

## Abstract

**Aims:**

To assess the cross-sectional association between exercise capacity, gas exchange efficiency and endothelial function, as measured by flow-mediated dilation (FMD) and nitroglycerin-mediated dilation (NMD) of the brachial artery, in a large-scale population-based survey.

**Methods:**

The study population was comprised of 1416 volunteers 25 to 85 years old. Oxygen uptake at anaerobic threshold (VO_2_@AT), peak exercise (peakVO_2_) and ventilatory efficiency (VE vs. VCO_2 _slope and VE/VCO_2_@AT) were assessed on a breath-by-breath basis during incremental symptom-limited cardiopulmonary exercise. FMD and NMD measurements at rest were performed using standardised ultrasound techniques.

**Results:**

Multivariable logistic regression analyses revealed a significant association between FMD and ventilatory efficiency in current smokers but not in ex-smokers or non-smokers. There was no association between FMD and VO_2_@AT or peak VO_2_. In current smokers, for each one millimetre decrement in FMD, VE/VCO_2_@AT improved by -3.6 (95% CI -6.8, -0.4) in the overall population [VE vs. VCO_2 _slope -3.9 (-7.1, -0.6)]. These results remained robust after adjusting for all major influencing factors. Neither exercise capacity nor ventilatory efficiency was significantly associated with NMD.

**Conclusion:**

In current smokers, FMD is significantly associated with ventilatory efficiency. This result may be interpreted as a potential clinical link between smoking and early pulmonary vasculopathy due to smoking.

## Introduction

Endothelial dysfunction represents an early, subclinical stage of vascular dysfunction that precedes the development of atherosclerosis [1] and predicts cardiovascular morbidity and mortality [2]. Its potential association with the functional capacity of the cardiovascular, pulmonary and muscular systems assessed by cardiopulmonary exercise testing (CPET) has been shown in small groups of young [3,4] and old, healthy individuals [5,6]. Usually, endothelial function is assessed by measuring flow-mediated dilation (FMD) using ultrasound. Occasionally, FMD is described in comparison to nitroglycerin-mediated dilation (NMD) as a surrogate of endothelial-independent vasoregulation. These measurements can be conducted in various vascular regions [7,8]; however, for feasibility reasons, this vascular response is commonly assessed in forearm vessels [9,10].

Dyspnoea, which is a symptomatic hallmark in patients with cardiovascular or pulmonary vascular diseases, can be quantified by gas exchange and ventilatory efficiency [11]. An impaired ventilatory efficiency is related to ventilation-perfusion inhomogeneities in patients with congestive heart failure [12,13] and pulmonary hypertension [14,15]. Thus, the ventilatory efficiency in eliminating carbon dioxide is considered a reliable measure for describing the relationship between pulmonary ventilation and perfusion [16]. Aside from the impact of cardiopulmonary diseases on ventilatory efficiency, previous studies have shown that smoking impairs ventilatory efficiency depending on the extent of cigarette exposure, which is possibly related to early airway dysfunction or, alternatively, pulmonary vasculopathy [17]. If endothelial function in the lungs mainly determines ventilatory efficiency, as assessed by gas exchange measurements, this would be a clinically accessible surrogate parameter to describe the functional integrity of pulmonary vessels and, hence, pulmonary perfusion. In normal pulmonary vessels, principal mediators of endothelial function, including nitric oxide (NO) and prostacyclin, regulate the maintenance of normal vascular tone and distribute the blood flow within the lung [18-20]. Correspondingly, diseases primarily affecting the pulmonary vascular bed, such as idiopathic pulmonary arterial hypertension, are associated with deficiencies in both mediators [21], which lead to diminished pulmonary endothelial function [22]. Previous data assessed within a small group of individuals suggest that the pulmonary vascular response to inhaled iloprost, a stable analogue of prostacyclin, is related positively to the extent of NO-dependent endothelial vasodilation, as assessed by FMD, in individuals with idiopathic pulmonary arterial hypertension (IPAH) [23]. It remains unknown whether FMD reflects endothelial dysfunction in pulmonary vessels in apparently healthy individuals as well.

Therefore, this investigation aimed to assess the potential link between peripheral endothelial function and gas exchange in a large-scale population-based study called the Study of Health in Pomerania (SHIP). The major hypothesis tested was that FMD is related to exercise capacity and ventilatory efficiency in a sample representing a wide age range of the general population.

## Methods

### Study population

The Study of Health in Pomerania (SHIP) is a population-based investigation in West Pomerania, a region in the northeastern part of Germany. The details of the study are given elsewhere [24,25]. In brief, a sample from a population aged 20 - 79 years was recruited from 1997 to 2001 to be evaluated during baseline SHIP-0. Between March 2002 and September 2006, the 5-year follow-up examinations (SHIP-1) were performed, which comprised 3300 participants (1711 women). The study was reviewed by a board of independent scientists and approved by the Ethics Committee of the University of Greifswald (approval number Dec 12, 2001: IIIUV73/01). All participants provided written informed consent.

We offered all SHIP-1 participants the opportunity to take part in measurements of endothelial function (FMD and NMD), body plethysmography and CPET. Of the 3300 SHIP-1 participants, 1705 volunteered in both CPET and endothelial function determination. We excluded 278 subjects with non-readable ultrasound images and 11 subjects with missing data. The study population available for the present analyses consisted of 1416 (701 men, 715 women) volunteers. Table 1 summarises the details of the study population.

**Table 1:** Descriptive statistics of the overall population (N = 1416).

	Study population			
	
	All (N = 1416)	Men (N = 701)	Women (N = 715)	p
Age^†^, years	52 (13.4)	53 (13.9)	51 (12.8)	< 0.01
Smoking, %				< 0.01
Non-smokers	43.0	29.3	56.4	
Ex-smokers	32.9	45.7	20.3	
Current smokers	24.2	25.0	23.4	
Physical activity	27.9	29.0	26.9	0.37
Height, cm	169.9 (9.0)	176.1 (6.6)	163.8 (6.6)	< 0.01
Weight, kg	80.4 (15.7)	87.5 (14.1)	73.4 (14.0)	< 0.01
O_2 _pulse	13.3 (3.5)	15.7 (3.1)	11.0 (2.0)	< 0.01
Heart rate at peak exercise	150.1 (23.2)	150.5 (23.9)	149.7 (22.5)	0.32
Peak VO_2_, ml/min	1983.8 (602.6)	2353.4 (592.9)	1621.5 (330.8)	< 0.01
VO_2_@AT, ml/min	1110.4 (307.6)	1261.2 (324.8)	962.6 (199.8)	< 0.01
VE vs. VCO_2 _slope	25.3 (4.2)	25.3 (4.4)	25.3 (3.9)	0.71
VE/VCO_2_@AT	27.5 (3.8)	27.8 (4.2)	27.2 (3.4)	0.07
Baseline BAD, mm	3.9 (0.7)	4.4 (0.5)	3.4 (0.4)	< 0.01
Post-occlusion BAD, mm	4.1 (0.7)	4.6 (0.5)	3.6 (0.4)	< 0.01
FMD, %	5.1 (3.9)	4.3 (3.2)	5.8 (4.3)	< 0.01
LDL cholesterol, mmol/l	3.5 (1.0)	3.5 (1.0)	3.5 (1.1)	0.27
Glucose, mmol/l	5.3 (1.2)	5.5 (1.3)	5.2 (1.1)	< 0.01

Concomitant medications:				
Antihypotensives	0.6	0.9	0.4	0.30
Peripheral vasodilators	0.9	1.3	0.4	0.08
Beta-blockers	20.8	20.8	20.8	1.00
Calcium channel blockers	7.3	8.4	6.2	0.10
Renin-angiotensin system interfering drugs	20.2	23.7	16.8	< 0.01
Non-steroidal anti-inflammatory drugs	8.7	5.7	11.6	< 0.01
Statins	11.0	13.0	9.0	0.02

Continuous data are expressed as the mean (± SD). Nominal data are given as percentages. *χ^2^-test (nominal data) or Kruskal-Wallis test (interval data). ^†^Age to CPET and endothelial function determination. peakVO_2_: peak oxygen; VO_2_@AT: oxygen uptake at anaerobic threshold: VE vs. VCO_2 _slope: ventilation to carbon dioxide output; VE/VCO_2_@AT: ventilatory efficiency; BAD: brachial artery diameter; FMD: flow-mediated dilation; and LDL: low-density lipoprotein.

For sensitivity analyses, an apparently healthy population without factors possibly interfering with endothelial function and CPET was defined. For this purpose, subjects with the following characteristics were excluded (overlaps exist): past myocardial infarction, echocardiographic evidence of ventricular dysfunction or valvular disease, electrocardiographic signs of ischaemia, neuromuscular or musculoskeletal disorders based on neurological examination, malignancies, pulmonary diseases, chronic obstructive bronchitis, bronchial asthma, drugs against obstructive airway disease including inhaled steroids [(ATC) code R03], arterial hypertension according to the definition of the World Health Organization[26] or the use of antihypertensive medications at the time of enrolment, and diabetes. Thus, the apparently healthy study population comprised of 985 volunteers (472 men, 513 women).

### Pre-exercise diagnostics and exclusion criteria

Sociodemographic and medical characteristics were assessed by computer-assisted personal interviews. Previous history of diseases was assessed based on self-reported physician's diagnosis. According to tobacco consumption, participants were categorised into current (one or more cigarettes per day), former, and non-smokers. Data on medication were collected using the anatomical therapeutic chemical (ATC) code [27]. Antihypotensives, antihypertensives, peripheral vasodilators, beta-blockers, calcium channel blockers, drugs acting on the renin-angiotensin system, statins, bronchodilators and nonsteroidal anti-inflammatory drugs (oral or inhaled), which could act as confounders, were included in the analyses. The diagnosis of arterial hypertension and diabetes mellitus was based on self-reported physician's diagnosis and physical examination [26].

### Flow- and nitroglycerin-mediated dilation

Endothelial function was assessed by FMD and endothelial-independent vasoregulation by NMD. Examinations were performed in a supine position by two observers. The subject's right arm was comfortably immobilised, and the brachial artery diameter was recorded 3-7 cm above the antecubital fossa using a 10-MHz linear array transducer ultrasound system (Cypress, Siemens AG, Erlangen, Germany). After the resting scan, a pneumatic cuff placed around the forearm 10 cm distal to the ultrasound location was inflated above a pressure of 220 mmHg for 5 min. Diameter measurements were repeated 60 s after cuff deflation. FMD was expressed as the post-occlusion brachial artery diameter corrected for baseline artery diameter (BAD) and as the ratio between brachial diameters before and after inflation of the pneumatic cuff. NMD was taken 3 min after sublingual administration of nitroglycerin (400 μg) in 1096 subjects (465 women). Examinations were performed and read by two observers. All ultrasound measurements in SHIP use strict quality management [28]. Intrareader, intraobserver, inter-reader, and interobserver variability were evaluated in certification procedures. Before data collection, 25 images were measured twice by each participating reader, and 12 volunteers were examined twice by each participating observer. During the data collection, observer certification procedures were repeated semiannually in six volunteers. At least 24 h was required between the two readings and examinations. Readers rated the quality of the digitally stored images as excellent, good, or adequate. The applied quality measures have been described elsewhere in detail [9].

### Exercise testing

CPET was performed with a physician in attendance according to a modified Jones protocol [29] using a calibrated electromagnetically braked cycle ergometer (Ergoselect 100, Ergoline, Germany). Protocol details are given elsewhere [17,30]. Gas exchange and ventilatory variables were analysed breath-by-breath using a VIASYS HEALTHCARE system (Oxycon Pro, Rudolph's mask), which had been recalibrated prior to each test. Twelve-lead ECGs were recorded at rest and every minute thereafter. Pulse oximetry was monitored continuously, and blood pressure was obtained by a cuff sphygmomanometer every two minutes. Prior to CPET, subjects were encouraged to reach maximal exhaustion. During exercise, no further motivation was utilised.

The minute ventilation, tidal volume, VO_2 _and VCO_2 _were acquired on a breath-by-breath basis and averaged over 10-second intervals. The peak oxygen uptake was defined as the highest 10-second average of VO_2 _in late exercise. The peak heart rate was averaged over that same period, and the peak O_2 _pulse was calculated as peak VO_2 _divided by peak heart rate. The peak respiratory exchange rate (RER) was calculated as the ratio of peak carbon dioxide output (VCO_2_) to peakVO_2_. The anaerobic threshold (AT) was determined according to Wasserman et al. [16]. The VE/VCO_2_@AT was averaged over a 30-second period. The VE/VCO_2 _ratio at rest was averaged over the last 30 seconds of a 3-minute resting period. The delta of the rest to anaerobic threshold VE/VCO_2 _ratio was calculated.

### Statistical analysis

Continuous data are expressed as the mean (± SD), and nominal data are expressed as numbers (percentages) and 95% confidence intervals. For bivariate statistics, the Mann Whitney *U *test (continuous data) and χ^2 ^test (nominal data) were applied to compare men and women. Multivariable linear regression models were performed to estimate the independent association of FMD or NMD with ventilatory efficiency and exercise capacity separately in current and non-/or ex-smokers in the overall and healthy populations for both sexes. Sensitivity analyses were performed to identify possible interfering factors. In the final model, we only considered those characteristics as confounders if inclusion in the model led to ≥ 10% change in the coefficient of interest. For this, clinical (medications against cardiopulmonary disorders, smoking, sex, age, height, weight, and arterial hypertension) and laboratory variables (diabetes and serum cholesterol) were included [9]. Thereafter, variables on the medications listed in Table 1 were entered into the model in various orders. Based on those analyses, the full models were adjusted for age, vascular baseline diameter, weight, and height. Statistical significance was defined by p < 0.05. All statistical analyses were performed with SAS software, version 9.1 (SAS Institute, Inc., Cary, NC, USA).

## Results

In the entire study population, the quality of FMD images was rated as excellent in 164 subjects (11.6%), good in 744 (52.5%), and adequate in 508 (35.9%). In the overall study population, the median RER at peak exercise was 1.10 (CI 1.05, 1.17) in men and 1.13 (CI 1.05, 1.19) in women. Thirty-five subjects reported a prior myocardial infarction, 20 had electrocardiographic evidence of myocardial ischaemia, 40 subjects had echocardiographic evidence of aortic dysfunction, 14 had echocardiographic evidence of mitral valve dysfunction, 30 echocardiographic had evidence of left ventricular dysfunction, 39 reported chronic obstructive pulmonary disease (COPD), 5 reported asthma and 37 reported other pulmonary diseases (overlaps existed). Use of drugs against cardiopulmonary diseases was reported by 187 subjects. None of the subjects revealed signs of pulmonary hypertension or had evidence of pulmonary embolism or clinically significant peripheral arterial vasculopathy.

Independent of smoking status in the healthy and overall population, FMD and NMD did not reveal any association with exercise capacity, as quantified by peakVO_2 _and VO_2_@AT, or with oxygen pulse.

In current smokers, FMD was inversely related to ventilatory efficiency (Table 2). In current smokers, for each one millimetre decrement in FMD, VE/VCO_2_@AT improved by -3.6 (95%CI -6.8; -0.4) in the overall population [VE vs. VCO_2 _slope -3.9 (-7.1, -0.6)] and -4.6 in apparently healthy volunteers (CI -8.2; -1.0) [VE vs. VCO_2 _slope -5.3 (-8.9, -1.7)]. In non- and ex-smokers FMD did not show any significant association with parameters of ventilatory efficiency (Table 3). NMD did not show a significant association to ventilatory efficiency. The decline in VE/VCO_2 _ratio from rest to exercise at the anaerobic threshold was not significantly associated with FMD. All effects were consistently reproducible through all reported or diagnosed comorbidities. There were no detectable differences between women and men.

**Table 2:** Association of flow-mediated dilation (assessed as post-occlusion brachial artery diameter corrected for baseline diameter) and nitroglycerin-mediated dilation with gas exchange and exercise capacity parameters in current smokers.

	Overall population					Healthy population				
	
	Peak VO_2_	VO_2_@AT	O_2 _pulse	VE vs. VCO_2 _slope	VE/VCO_2_@AT	Peak VO_2_	VO_2_@AT	O_2 _pulse	VE vs. VCO_2 _slope	VE/VCO_2_@AT
	
	β coefficient (95% CI)	β coefficient (95% CI)	β coefficient (95% CI)	β coefficient (95% CI)	β coefficient (95% CI)	β coefficient (95% CI)	β coefficient (95% CI)	β coefficient (95% CI)	β coefficient (95% CI)	β coefficient (95% CI)
**FMD**										
Adjusted for baseline BAD	650.0 (243.5; 1056.5)	288.7 (89.8; 487.5)	2.7 (0.4; 5.0)	-5.2 (-8.5; -1.8)	-5.2 (-8.5; -1.8)	866.8 (426.7; 1306.9)	314.2 (90.5; 538.0)	3.1 (0.5; 5.6)	-7.0 (-10.6; -3.3)	-6.5 (-10.2; -2.8)
Fully adjusted^†^	35.2 (-261.2; 331.6)	57.0 (-117.9; 231.9)	-0.1 (-2.0; 1.8)	-3.9 (-7.1; -0.60)	-3.6 (-6.8; -0.4)	109.0 (-227.6; 445.6)	59.1 (-147.2; 265.4)	-0.2 (-2.3; 1.9)	-5.3 (-8.9; -1.7)	-4.6 (-8.2; -1.0)
**NMD**										
Adjusted for baseline BAD	448.6 (180.1; 717.1)	93.7 (-38.4; 225.8)	1.0 (-0.5; 2.6)	-0.8 (-2.9; 1.3)	-1.4 (-3.4; 0.6)	349.3 (52.0; 646.6)	50.9 (-98.0; 199.9)	0.6 (-1.1; 2.3)	-0.5 (-2.9; 1.8)	-1.1 (-3.4; 1.2)
Fully adjusted^†^	-2.1 (-203.1; 199.0)	-19.2 (-138.1; 99.6)	-0.6 (-1.9; 0.7)	0.6 (-1.5; 2.7)	-0.2 (-2.2; 1.8)	-17.3 (-241.4; 206.7)	-27.9 (-166.1; 110.2)	-0.7 (-2.1; 0.7)	0.9 (-1.4; 3.2)	0.2 (-2.0; 2.4)

^†^Age to CPET, endothelial function determination and baseline brachial artery diameter, height and weight.

Peak VO_2_: peak oxygen uptake; VO_2_@AT: oxygen uptake at anaerobic threshold; O_2 _pulse: peak oxygen pulse; VE vs. VCO_2 _slope: ventilation to carbon dioxide output; VE/VCO_2_@AT: ventilatory equivalent at anaerobic threshold; and BAD: post-occlusion brachial artery diameter.

**Table 3:** Association of flow-mediated dilation (assessed as post-occlusion brachial artery diameter corrected for baseline diameter) and nitroglycerin-mediated dilation with exercise capacity parameters in non-smokers and ex-smokers.

	Overall population					Healthy population				
	
	Peak VO_2_	VO_2_@AT	O_2 _pulse	VE vs. VCO_2 _slope	VE/VCO_2_@AT	Peak VO_2_	VO_2_@AT	O_2 _pulse	VE vs. VCO_2 _slope	VE/VCO_2_@AT
	
	β coefficient (95% CI)	β coefficient (95% CI)	β coefficient (95% CI)	β coefficient (95% CI)	β coefficient (95% CI)	β coefficient (95% CI)	β coefficient (95% CI)	β coefficient (95% CI)	β coefficient (95% CI)	β coefficient (95% CI)
**FMD**										
Adjusted for baseline BAD	1027.0 (777.0; 1276.9)	357.0 (223.6; 490.4)	3.7 (2.4; 5.0)	-3.3 (-5.3; -1.4)	-3.1 (-4.8; -1.4)	987.9 (694.1; 1281.7)	353.0 (189.4; 516.6)	3.5 (2.0; 5.0)	-3.5 (-5.8; -1.2)	-2.8 (-4.9; -0.8)
Fully adjusted^†^	-33.8 (-214.9; 147.4)	-18.7 (-141.6; 104.3)	-0.4 (-1.5; 0.7)	-0.3 (-2.2; 1.6)	-0.5 (-2.2; 1.2)	78.9 (-140.2; 297.9)	57.8 (-96.9; 212.5)	-0.1 (-1.3; 1.1)	-1.1 (-3.5; 1.2)	-0.8 (-2.8; 1.2)
**NMD**										
Adjusted for baseline BAD	914.3 (745.4; 1083.2)	285.6 (194.3; 377.0)	3.2 (2.3; 4.2)	-2.0 (-3.3; -0.7)	-1.6 (-2.7; -0.4)	801.6 (588.6; 1014.6)	181.3 (61.4; 301.3)	3.4 (2.3; 4.5)	-1.8 (-3.4; -0.1)	-1.0 (-2.5; 0.5)
Fully adjusted^†^	135.7 (10.6; 260.8)	2.5 (-82.7; 87.7)	0.1 (-0.6; 0.9)	0.5 (-0.8; 1.8)	0.5 (-0.6; 1.7)	88.9 (-69.5; 247.5)	-79.8 (-192.1; 32.5)	0.3 (-0.6; 1.2)	0.2 (-1.5; 1.9)	0.8 (-0.7; 2.3)

^†^Age to CPET, endothelial function determination and baseline brachial artery diameter, height and weight.

Peak VO_2_: peak oxygen uptake; VO_2_@AT: oxygen uptake at anaerobic threshold; O_2 _pulse: peak oxygen pulse; VE vs. VCO_2 _slope: ventilation to carbon dioxide output; VE/VCO_2_@AT: ventilatory equivalent at anaerobic threshold; BAD: post-occlusion brachial artery diameter.

## Discussion

In terms of this study's hypotheses, neither in smokers nor non-smokers did endothelial function reveal any association with peak exercise capacity as verified by peak VO_2 _or aerobic exercise capacity, as judged by VO_2_@AT. Thus, it has to be postulated that NO-dependent endothelial function plays a minor, unverifiable role in muscle endurance and exercise capacity as assessed within a symptom-limited CPET in healthy volunteers.

Previous studies have suggested a potential interference of endothelial functioning with exercise capacity [3-6]. Palmieri et al. have shown a tight correlation between VO_2_@AT and peak VO_2 _in FMD in young adults [3], which is comparable to results that have been reported in older individuals by Rinder et al. and Rywik et al. [5,6]. Furthermore, exercise training seems to influence endothelial function with corresponding increases in exercise capacity [31], and training status has been shown to influence exercise capacity and endothelial function [4]. However, all of these studies were based on small groups of volunteers and do not represent a general population. The suggested impact of NO-dependent endothelial function on exercise capacity is now challenged by our results. According to the data presented here, endothelial dysfunction quantified by FMD has no significant impact on exercise capacity as quantified by oxygen uptake at anaerobic threshold or peak exercise and is independent of smoking status and potentially confounding diseases. To what extent exercise endurance training may influence FMD parallel to exercise capacity could not be investigated by our study and has to be addressed by longitudinal and interventional studies.

This study shows that in current smokers, FMD is significantly correlated to ventilatory efficiency independent of sex and co-morbidities. This correlation is not verifiable in non- or ex-smokers. To the best of our knowledge, this is the first study describing a correlation between NO-dependent endothelial function and gas exchange efficiency and exercise capacity in a large-scale population-based study. Our previous work assessed the influence of smoking on exercise capacity and gas exchange efficiency in the same population-based study [17]. In that study, ventilatory efficiency correlated with the extent of smoking in individuals without apparent cardiovascular or pulmonary diseases and with normal lung function, body plethysmography and echocardiography [17]. One aspect of that study was to interpret changes in ventilatory efficiency as an early marker of parenchymal or vascular lung disease related to smoking independent of lung function abnormalities. Based on the inverse relationship between NO-dependent endothelial function and gas exchange efficiency, a vascular hypothesis might be supported. Endothelial dysfunction is related to several peripheral vascular diseases, such as arterial hypertension, diabetic vasculopathy [31-33] and pulmonary vascular diseases [18,22,23,34]. However, ventilatory efficiency is impaired in patients with abnormal pulmonary circulation and reliably mirrors the severity of pulmonary vascular diseases, such as pulmonary arterial hypertension [35]. The significant correlation between ventilatory efficiency and FMD independent of health status may potentially suggest a sub-clinical smoking-related pulmonary vascular abnormality. Smoking has been proposed to potentially trigger pulmonary vascular disease in experimental studies in animals [36,37]. In addition, smoking has been discussed as an important contributor to the development of pulmonary hypertension in COPD patients [38]. Pulmonary vascular abnormalities in patients with mild-to-moderate COPD mainly consist of the thickening of the intima in pulmonary muscular arteries, which interferes with lumen size [38]. Interestingly, studies conducted in smokers with normal lung function have also revealed intimal thickening in pulmonary muscular arteries [39]. In addition, ventilatory efficiency and gas exchange may be impacted by early airway disease as well. The potential link between smoking, early airway disease and pulmonary vasculopathy may be due to low-grade systemic inflammation. In early stages of chronic obstructive pulmonary disease, perfusion heterogeneity and low airflow obstruction have been observed, which suggests that in smokers, initially the smallest airways, parenchyma, and pulmonary vessels are affected [40]. In contrast to FMD, NMD is a marker of endothelium-independent vasodilation [41]. Although there was an association between smoking and FMD in our study, we did not find such an association for NMD. This result strengthens the hypothesis that smoking may affect endothelial function via the NO system.

Finally, our study has limitations. The SHIP project, as a large-scale observational population-based study, was not designed to test the hypotheses that vascular abnormalities are related to ventilatory inefficiency in smokers. However, to the best of our knowledge, this is the first study to describe the interaction of endothelial function, exercise capacity and ventilatory efficiency in a large population sample. Because this study is based on individual volunteering, as in any population-based cross-sectional survey, we cannot fully rule out selection bias. We observed that CPET volunteers were younger than non-participants, which might have led to a healthier study population [42].

Furthermore, due to ethical reasons the design of a population-based survey does not allow for histopathological investigations. Thus, the final proof of the hypotheses discussed here is pending.

## Conclusions

In conclusion, in a general adult population, peripheral NO-dependent vasodilation assessed by FMD was not associated with exercise capacity and was independent of coexisting diseases. A significant, inverse association between FMD and ventilatory efficiency did exist in smokers, whereas this association was not verifiable in non- or ex-smokers. In current smokers, a decreased FMD was associated with impaired ventilatory efficiency. This association may be interpreted as a potential link between smoking and early pulmonary vasculopathy due to smoking exposure.

## Abbreviations

AT: Anaerobic threshold; ATC code: Anatomical-technical-chemical code; BAD: Brachial artery diameter; CPET: Cardiopulmonary exercise testing; ECG: Electrocardiogram; FMD: Flow-mediated dilation; IPAH: Idiopathic pulmonary arterial hypertension; NMD: Nitrogen-mediated dilation; NO: Nitrate oxide; peakVO2: Peak oxygen uptake; RER: Respiratory exchange rate; SHIP: Study of Health in Pomerania; VCO2: Carbon dioxide output; VE vs. VCO2 slope: Slope of the regression of minute ventilation to carbon dioxide output; VE/VCO2@AT: Minute ventilation to carbon dioxide ratio at anaerobic threshold; VO2: Oxygen uptake; VO2@AT: Oxygen uptake at anaerobic threshold.

## Competing interests

The authors declare that they have no competing interests.

## Authors' contributions

SG drafted the manuscript, made substantial contributions to the conception, design and acquisition of the data as well as the analysis and interpretation of the data and has given final approval for the version to be published. AO acted as one of the leading statisticians that analysed the data, made substantial contributions to the conception, design and acquisition of the data, was involved in drafting the manuscript and revising it critically for important intellectual content and has given final approval for the version to be published. CFO, SBF, KE, CS, RE, MD, HV and BK have made substantial contributions to the conception, design and acquisition of the data as well as the analysis and interpretation of the data, were involved in drafting the manuscript or revising it critically for important intellectual content and have given final approval for the version to be published.
